# Potential characterization of yeasts isolated from Kazak artisanal cheese to produce flavoring compounds

**DOI:** 10.1002/mbo3.533

**Published:** 2017-12-26

**Authors:** Xiaoji Zheng, Kaixiong Li, Xuewei Shi, Yongqing Ni, Baokun Li, Bin Zhuge

**Affiliations:** ^1^ The Key Lab of Industrial Biotechnology of Ministry of Education Research Centre of Industrial Microorganisms School of Biotechnology Jiangnan University Wuxi Jiangsu Province China; ^2^ College of Food Sciences Shihezi University Shihezi Xinjiang Uighur Autonomy Region China

**Keywords:** Hazak artisanal cheese, volatile compounds, yeast microflora

## Abstract

Cheese is a typical handcrafted fermented food in Kazak minority from the Uighur Autonomy Region in China and Central Asia. Among the microbial community that is responsible for Kazak cheese fermentation, yeasts play important role in flavor formation during ripening. To develop ripening cultures, we isolated 123 yeasts from 25 cheese products in Kazak, and identified 87 isolates by the D1/D2 domain of the large subunit rRNA gene sequence. *Pichia kudriavzevii* was the dominant yeast in Kazak cheese, followed by *Kluyveromyces marxianus* and *Kluyveromyces lactis*. Of these, the ability to exhibit enzyme of dominant isolates and contribution to the typical flavor of cheeses was assessed. Enzyme producing yeast strains were inoculated in Hazak cheese‐like medium and volatile compounds were identified by head space solid phase micro extraction coupled to gas chromatography and mass spectroscopy. *Pichia* *kudriavzevii* N‐X displayed the strongest extracellular proteolytic and activity on skim milk agar and produced a range of aroma compounds (ethanol, ethyl acetate, 3‐methylbutanol, and acetic acid) for Kazak cheese flavor, could be explored as ripening cultures in commercial production of Kazak cheeses.

## 1 INTRODUCTION

Kazak cheese is a typical handcrafted fermented food in Central Asia, also in Uighur Autonomy Region, China. Kazak artisanal cheese has been produced through empirical trials. Spontaneous fermentation, using old yogurt as starter or no starter, takes place in goatskin bags, and involves beating with wooden stick to promote fermentation. Fresh cheese is placed on a bamboo board for 30–90 days of spontaneous ripening, at an average temperature between 5°C and 10°C with a humidity of 85% to 90%. Because of using complex microbial communities, the flavor of traditional handmade cheese is richer than that of industrially produced cheese which uses lactic acid bacteria as starter cultures. However, the traditional artisanal methods of making various cheese have been replaced by industrial production processes along with decline of Kazak nomadic customs. The microorganisms contribute to cheese flavor through their lysis and the release of intracellular enzymes which mainly determine the development of flavor during the cheese ripening (Fasoli et al., 2015; Padilla, Belloch, López‐Díez, Flores, & Manzanares, 2014; Pescuma, de Valdez, & Mozzi, 2015).

Ripening is crucial for cheese quality and flavor. Yeasts possess efficient proteolytic and lipolytic enzymatic systems, which contribute to the formation of aromatic compounds during ripening (McSweeney, 2004; Padilla et al., 2014). Among them, *Debaryomyces hansenii* is the major yeast species found in most surface‐ripened cheeses, and it can utilize lactose, lactic, and citric acids for its proliferation (Breuer & Harms, 2006; Cano‐García, Flores, & Belloch, 2013). *Kluyveromyces lactis* and *Kluyveromyces marxianus* are dairy relevant yeast species and have lactose‐fermenting and cell growth ability in cheese (Leclercq‐Perlat, Corrieu, & Spinnler, 2004). Moreover, *Yarrowia lipolytica* and *Geotrichum candidum* have an important effect on the flavor of cheese and dairy products (Steensels & Verstrepen, 2014).

Previous studies on cheese complex microbiota have focused on metagenomic and metabolomics analysis of a ripened cheese, and reflects the microbial complexity of dairy products (Escobar‐Zepeda, Sanchez‐Flores, & Baruch, 2016; Pisano, Scano, Murgia, Cosentino, & Caboni, 2016; Wolfe, Button, Santarelli, & Dutton, 2014), but few studies have determined potential correlations between metabolites produced and contribution of predominant microorganisms present in cheese (De Pasquale, Di Cagno, Buchin, De Angelis, & Gobbettia, 2014b; De Pasquale et al., 2014a; Wang, Lu, Shi, & Xu, 2016; Wang et al., 2017).

Due to different geographical and climatic conditions, the sensory characteristics and quality of Kazak cheese are not easily controlled. Therefore, it is important to define the mechanisms involved in microbial flavor formation in traditional cheese (Binetti, Carrasco, Reinheimer, & Suarez, 2013) and examine relationships between microbial communities and metabolic capacity (Ceugniez, Drider, Jacques, & Coucheney, 2015). In the present study, the diversity of culturable yeast was examined and the effect of enzyme producing yeasts on flavor of Kazak cheese was investigated. This research could be used to develop new starter or adjunct cultures to improve cheese flavor during ripening.

## 2 MATERIALS AND METHODS

### 2.1 Cheese samples

Twenty‐five home‐made cheese samples were collected from different local ethnic minority farmhouses from the following Kazak districts in China: Ili (5 cheeses), Tacheng (8), Altay (4), Barkol Kazak Autonomous County (4) and Mori Kazak Autonomous County (4), and samples were stored at 4°C before analysis.

### 2.2 Yeasts isolation

A cheese sample (10 g) was aseptically taken from the rind surface and curd as described by Wolfe et al. (2014). The sample was transferred into tubes containing 50 ml of sterile 0.9% sodium chloride solution, and the mixture was homogenized on a shaker (SPH‐1112D, Shanghai Shiping Laboratory Equipment Co., Ltd, Shanghai, China) for 30 min at 20°C. Samples were serially diluted and aliquots (100 μl, in triplicate) of each dilution were surface spread on Rose Bengal Chloramphenicol Agar (RBCA) (Shanghai Sangon Biological technology Co., LTD, Shanghai, China) containing 200 mg·L^−1^ of chloramphenicol. Following incubation for 5 days for enumeration and isolation at 28°C (Atanassova et al., 2016), and yeast colonies that showed different morphologies were isolated from RBCA plates and were stored in Yeast Extract Peptone Dextrose Medium containing 20% glycerol at −80°C.

### 2.3 Identification of yeast isolates

Yeast isolates were identified by combining the results from analysis of physiological and biochemical characteristics, and sequencing of the D1/D2 domain of the large subunit rRNA gene. The D1/D2 domain of the large subunit rRNA gene was amplified using primers NL1 (5ʹ‐GCATATCAATAAGCGGAGGAAAAG‐3ʹ) and NL4 (5ʹ‐TCCTCCGTCTATTGATATGC‐3ʹ). DNA amplification was performed with the following PCR program: (1) initial denaturing step at 95°C for 5 min; (2) 36 cycles of 40 s at 94°C, 40 s at 55°C and 30 s at 72°C; (3) final extension step at 72°C for 10 min. Amplification products were separated by electrophoresis in a 1.2% w/v agarose gel. Sequences were obtained with an Applied Bio systems DNA Sequencer, mod. ABIPRISM 377 (Shanghai Sangon Biological technology Co., LTD, Shanghai, China). Strains were identified by comparing sequences in the GenBank database (BLASTN freeware from http://www.ncbi.nlm.nih.gov/BLAST).

### 2.4 Screening of the protease, lipase, and β‐galactosidase producing yeast strains

A set of 87 yeast isolates were tested for the ability to produce protease, lipase and β‐galactosidase. To screen for β‐galactosidase activity, 10 μl of 48 hr yeast cultures (YPD broth, 30°C, cell concentration of about 10^6^ cfu/ml) were used to inoculate on YPD plates supplemented with 0.1 mg·ml^−1^ 5‐bromo‐4‐chloro‐3‐indolyl β‐D‐galactopyranoside (X‐gal) (Shanghai Sangon Biological technology Co., LTD, Shanghai, China). The plates were incubated at 28°C for 48–120 hr and positive colonies with β‐galactosidase activity appeared blue (Nakagawa et al., 2006).

For determination of intracellular β‐galactosidase activity, the method of Jochems was used with some adjustments (Jochems et al., 2011). Enzyme solution (100 μl) was added to 900 μl of a 5 mmol/L *o*‐nitrophenyl‐β‐D‐galactopyranoside substrate solution (Shanghai Sangon Biological technology Co., LTD, Shanghai, China). Tubes were incubated in a water bath for 10 min at 37°C. The reaction was stopped by adding 1 ml of a 1 mol/L sodium carbonate solution and the tubes remained at 37°C for 5 min. The samples were then measured in an ultraviolet spectrophotometer (UV‐1750, Shimadzu Corporation, Kyoto, Japan) at 420 nm (Wang, Li, Yu, Zhang, & Liu, 2010). One unit of enzyme activity was defined as the amount of enzyme that liberated 1 μmol/L of *o*‐nitrophenol (Shanghai Sangon Biological technology Co., LTD, Shanghai, China) in 1 min at 37°C.

The ability to degrade protein was assessed on skim milk agar (consisted of 10 g·L^−1^ skim milk and 20 g·L^−1^ agar, final pH 6.5) and casein agar (containing 20 g·L^−1^ of casein solution, pH 5.5). A sample of 10 μl yeast cultures (YPD broth, 30°C, cell concentration of about 10^6^ cfu/ml) were used to inoculate on two different media, after 48–120 hr incubation at 28°C, the ability to degrade protein was indicated by a precipitate (opaque halo) around each colony after incubation at 28°C for 48–120 hr (Cardoso et al., 2015).

Selected yeasts were also screened for lipolytic activities on two different media. The first was cultivated on tributyrin agar containing 10 ml tributyrin, 5 g peptone, 20 g glucose, 10 g yeast extract and 20 g agar L^−1^. A sample of 10 μl of yeast cultures (YPD broth, 30°C, cell concentration of about 10^6^ cfu/ml) were used to inoculate on the corresponding medium, and the presence of a clear halo around the colonies indicated a positive reaction after 24–48 hr of incubation at 28°C (Kumar et al., 2012; Padilla et al., 2014).

The second medium was Rhodamine olive oil agar medium (ROA). The growth medium contained (g·L^−1^): 1.0 g K_2_HPO_4_, 1.0 g (NH_4_)_2_SO_4_, 0.01 g FeSO_4_·7H_2_O, 0.5 g MgSO_4_·7H_2_O, 5.0 g yeast extract, 20 g agar, 120 ml olive oil solution, and 10 ml 0.05% Rhodamine B, (final pH 7.0). The olive oil solution was prepared by mixing 100 ml olive oil and 300 ml 2% polyvinyl alcohol (dissolved in deionized water and heated in a microwave) with vigorous stirring. A preculture of yeast in YPD was grown at 28°C for 48 hr (cell concentration of about 10^6^ cfu/ml), and 1 μl culture was inoculated in an oxford cup on the ROA medium and cultivated at 28°C for 48 hr. The lipolytic activities was observed as a clear fluorescent zone and transparent halo around each colony in the ROA medium by ultraviolet light (Dong et al., 2016; Kumar et al., 2012). Extracellular proteolytic and lipolytic activities of the selected yeasts were calculated by measurement of the hydrolysis or precipitation zone.

### 2.5 Imitation of Hazak cheese‐like medium and inoculation of enzyme producing yeast strains

Unsalted, unripened Hazak cheese were chosen from local ethnic minority farmhouses. Cheese of 200 g was added to 20 ml of sterile demineralized water contain NaCl (20 g) and stirred for 5 min using a hand‐blender. The mixture was sterilized for 20 min at 121°C. After cooling to 28°C–30°C, cheese was inoculated with each enzyme producing yeast strain (cell concentration of approximately 1.5 × 10^6^−1.8 × 10^6^ cfu/ml), then the inoculated cheese were hand‐shaped to round blocks and incubated for 12 days in incubator (AHWS‐150L, Changzhou Aihua instrument manufacturing co., LTD, Jiangsu, China) at 15°C with a humidity of 85%. A blank uninoculated cheese was incubated under the same conditions.

### 2.6 Analysis of volatile compounds from enzyme producing yeast strains

The production of volatile compounds from species *K. marxianus*,* Pichia kudriavzevii*, and *K. lactis* which had the ability to produce protease, lipase, and β‐galactosidase enzymes was determined. Five grams of cheese containing 2 μl of the internal standard solution containing 0.0555 mg·ml^−1^ of *p*‐dichlorobenzene (Shanghai Sangon Biological technology Co., LTD, Shanghai, China) was transferred into a 20 ml headspace bottle using head space solid phase micro extraction (HS‐SPME) analysis with a 2 cm fiber and 75 μm divinylbenzene/carboxen on polydimethylsiloxane after aging. The SPME fiber was exposed to the headspace and adsorbed at 50°C for 30 min in a water bath and inserted into the injection port of the GC‐MS (SCION SQ 456‐GC, BRUKER, Massachusetts, America) for thermal desorption at 250°C for 10 min. Separation of compounds was performed on a DB‐WAX column (30 m × 0.25 mm × 0.25 μm film thickness, Agilent Technologies). Helium (purity = 99.999%) was set as a carrier gas with a flow rate of 0.8 ml·min^−1^. The column temperature was maintained at 40°C for the first 3 min, then increased to 90°C at the rate of 5°C min^−1^ and finally raised at a rate of 10°C min^−1^ to 230°C for 7 min. Injector and detector temperatures were set to 250°C. The mass spectra were operated under the electron ionization mode at 70 eV and data were acquired in a scanning mode across the range 30–450 amu. The volatile compounds were identified by comparison with the retention indices (RI) of commercial references compounds provided by Sigma‐Aldrich (St. Louis, MO), or comparison with mass spectra in the Wiley and NIST libraries. N‐alkanes (C5–C30, Sigma R‐8769) were analyzed under the same chromatographic condition to calculate retention indices (RI) for the volatile compound (Delgado, González‐Crespo, Cava, García‐Parra, & Ramírez, 2010; Garabal, Rodriguez‐Alonso, Franco, & Centeno, 2010).

### 2.7 Statistical evaluation

The effect of selected yeast strains on volatile compounds was analyzed using analysis of variance, where Duncan's test was used to determine differences among distinct groups. Statistical procedures were performed with the SPSS (version 15.0). Principal component analysis (PCA) was performed by Unscramble software (V.9.7, CAMO ASA, Oslo, Norway) to examine relationships between yeast species and the main volatile compounds produced by each strain. Heat maps were formed and permutation analyses of volatile components were used for clustering by R, version 3.3.2.

## 3 RESULTS AND DISCUSSION

### 3.1 Yeast identification and origin

A total of 123 yeast isolates were obtained from 25 types of cheese in China (Figure S1), and 87 isolates were identified using biochemical tests and the D1/D2 domain of the large subunit rRNA gene sequence analysis. The isolated strains were classified into eight genera including, *P. kudriavzevii*,* K. marxianus*,* K. lactis*,* Clavispora lusitaniae*,* Lodderomyces elongisporus*,* Candida parapsilosis, Galactomyces geotrichum*, and *Pichia fermentans*.

*Pichia kudriavzevii* represented approximately 52% of the yeasts identified, and was the dominant yeast in Kazak cheese, followed by *K. marxianus* and *K. lactis* (Table 1). In 1998, Kurtzman transferred the *Issatchenkia orientalis* to *Pichia* under a new species, namely *P. kudriavzevii*, and *P. kudriavzevii* was present in traditional fermented such as Armada cheese, Kefir, and acid curd cheese which plays a role in flavor production (Yadav, Bezawada, Yan, Tyagi, & Surampalli, 2012). *Pichia kudriavzevii* is the major yeast species found in Kazak cheese, due to its ability tolerance to cheese environment such as low pH, high NaCl concentration, and lactate. *Debaryomyces hansenii* was the most predominant yeast species isolated from both the inside and outside of the cheeses, followed by *Y. lipolytica* and *K. lactis* (Ozturkoglu‐Budak, Wiebenga, Bron, & de Vries, 2016a). The prevalent yeast species found in Kazak artisanal cheese were different from those reported in other types of cheese (Atanassova et al., 2016; Chebenova‐Turcovska, Zenisova, Kuchta, Pangallo, & Brezna, 2011; Cogan et al., 2014), and this is likely to be attributed to different environmental factors and processing technology.

**Table 1:** Species of the yeast isolates obtained from the predominant Kazak areas of 25 raw cow's milk cheeses made in North of Uighur Autonomous Region, China

Yeast species	Assignation to species level^a^	Cheese source^b^					Total number of isolates
		A	I	T	B	M	
*Kluyveromyces marxianus*	SEQ + PBC	3	4	4	2	3	16
*Kluyveromyces lactis*	SEQ + PBC	2	3	2	1	2	10
*Pichia kudriavzevii*	SEQ + PBC	5	12	8	6	10	41
*Clavispora lusitaniae*	SEQ + PBC	0	2	3	0	2	7
*Candida parapsilosis*	SEQ + PBC	1	0	1	1	0	3
*Galactomyces geotrichum*	SEQ + PBC	0	2	1	0	0	3
*Pichia fermentans*	SEQ + PBC	1	1	0	1	0	3
*Lodderomyces elongisporus*	SEQ + PBC	1	0	3	0	0	4
Unidentified		10	5	7	6	8	36
Total number of isolates		23	29	29	17	25	123

a PBC, physiological and biochemical characteristics; SEQ, Identification by the D1/D2 domain of the large subunit (LSU) rRNA gene sequence.

b A represented Cheese from Altay area; I, Ili Kazak Autonomous Prefecture; T, Tacheng area; M, Mulei Kazakh Autonomous County and B, Barkol Kazak Autonomous County, Uighur Autonomous Region, China.

*Kluyveromyces marxianus* and *Kluyveromyces lactis* are commonly found in naturally fermented cheese and dairy products (Binetti et al., 2013). *Kluyveromyces marxianus* contributes greatly to the typical flavor of traditionally produced cheese (Padilla et al., 2014; Sørensen, Gori, Petersen, Jespersen, & Arneborg, 2011), and could be further studied for its ability to produce flavor compounds in such products (Morrissey, Etschmann, Schrader, & de Billerbeck, 2015). *Geotrichum candidum* was also important in the initial stages of ripening of cheese (Cogan et al., 2014), and *K. lactis* and *G. candidum* were shown to improve the flavor of Camembert cheese (Fox, Guinee, Cogan, & McSweeney, 2017).

### 3.2 Enzymatic abilities of the yeast isolates

Some proteolytic and lipolytic yeasts could produce flavor compounds during ripening mainly through three pathways: (1) metabolism of lactose through β‐galactosidase, (2) lipolysis and fatty acid metabolism through lipase, and (3) proteolysis and amino acid metabolism by protease during cheese ripening (Cholet, Henaut, Casaregola, & Bonnarme, 2007; Fox, McSweeney, Uniacke‐Lowe, & O'Mahoney, 2015). These pathways involve relevant enzymes (β‐galactosidase, protease, and lipase), which catalyze a complex series of biochemical reactions and lead to desirable flavors in cheese. In this work, the intracellular β‐galactosidase, extracellular lipolytic (tributyrin, ROA medium) and proteolytic (skim milk and casein) enzyme activities of yeast isolates were examined.

Forty isolated yeasts exhibited lipase, protease and β‐galactosidase enzymatic activities. In this study, *K. marxianus* 24‐5 and *P. kudriavzevii* N‐X displayed lipase, protease, and β‐galactosidase activities (Figure S2). The highest intracellular β‐galactosidase activity (25.3 ± 0.009 U·ml^−1^) was produced by *K. marxianus* 24‐5. *Kluyveromyces marxianus* 27‐2 and *P. kudriavzevii* N‐X possessed high intracellular β‐galactosidase activities (Table 2, Figure S2). Most of the *K. marxianus* and *K. lactis* isolates had *LAC* 12 and *LAC* 4 genes, encoding a lactose permease and a β‐galactosidase (Morrissey et al., 2015), respectively, and had the ability to ferment lactose which may contribute to its growth in the initial stages of the ripening process (Jochems et al., 2011).

**Table 2:** GenBank accession, culture collection numbers and enzyme abilities of the yeast isolates from screening in this study

Strain No.	Closest known species (% similarity)	Culture collection number of NCBI	GenBank accession number (reference)	Origin	Protease (pH 6.0)		Lipase^d^		β‐Galactosidase	
					Skim milk^a^	Casein^b^	ROA^c^	TBA^d^	X‐gal^e^	activity^f^
5‐7	*Kluyveromyces marxianus* (99%)	MF461005	KY108103.1	Y	0.71 (0.49–0.95)	0.79 (0.53–1.00)	0.70 (0.52–0.73)	0.60 (0.55–0.80)	+	3.2 ± 0.024
5‐11	*Kluyveromyces marxianus* (100%)	MF461007	KY108106.1	Y	0.82 (0.63–0.95)	0.78 (0.62–0.90)	0.88 (0.65–0.95)	0.83 (0.76–0.85)	+	5.6 ± 0.004
N‐9	*Kluyveromyces marxianus* (99%)	MF461003	KY108101.1	M	1.00	0.95 (0.86–1.00)	1.00	0.88 (0.65–0.92)	+	6.2 ± 0.102
24‐5	*Kluyveromyces marxianus* (100%)	MF461002	KY108102.1	B	0.85 (0.75–1.00)	0.70 (0.60–0.92)	0.84 (0.72–0.88)	0.78 (0.64–0.82)	+++	25.3 ± 0.009
27‐2	*Kluyveromyces marxianus* (100%)	MF461006	KU687354.1 JF715181.1	Y	0.93 (0.85–1.00)	0.86 (0.60–1.00)	0.96 (0.90–1)	0.90 (0.84–1.00)	++	16.7 ± 0.036
NTE	*Kluyveromyces lactis* (99%)	MF461000	KY108046.1	Y	0.95 (0.9–1.00)	0.83 (0.65–0.90)	0.77 (0.70–0.81)	0.65 (0.50–0.95)	–	0
N‐S	*Kluyveromyces lactis* (99%)	MF461009	KY108048.1	B	0.96 (0.8–1.00)	1.00	0.70 (0.55–0.90)	0.78 (0.60–0.91)	–	0
N‐X	*Pichia kudriavzevii* (100%)	MF461008	KY108855.1	Y	0.64 (0.60–0.76)	0.53 (0.50–0.55)	0.62 (0.60–0.68)	(0.48–0.56) 0.78	+	10.8 ± 0.381
4‐5	*Pichia kudriavzevii* (100%)	MF461004	KY108855.1	Y	0.61 (0.54–0.70)	0.63 (0.5–0.72)	0.81 (0.75–0.92)	0.84 (0.62–1.00)	+++	20.6 ± 0.756
10‐8	*Pichia kudriavzevii* (99%)	MF461001	KY108849.1	B	0.86 (0.70–0.96)	0.75 (0.63–0.94)	0.83 (0.60–0.90)	0.72 (0.62–0.92)	–	0

NCBI, National Center of Biotechnology Information.

a Results expressed as hydrolysis zone (Hz) values in milk medium (mean values; ranges in brackets). Values lower than 0.95 are considered positive; values lower than 0.60 confirm strong positive reactions.

b Hydrolysis zone (Hz) values in Casein medium.

c The lipase activity was valued with fluorescent zone(Fz) values around the colony by ultraviolet light on Rhodamine olive oil agar medium (ROA).

d Tributyrin agar (TBA) was used to confirm positive yeast strains with lipolytic activities.

e A positive of strain was blue in YPD plates supplemented with 1 mmol/L IPTG and10 mg/ml X‐gal.

f Activity of β‐galactosidase from selected yeasts strains.

The yeast isolates with the highest extracellular proteolytic activities on skim milk agar were *P. kudriavzevii* N‐X (mean of hydrolysis zone value (Hz) of 0.64) and *P. kudriavzevii* 4‐5 (mean Hz value of 0.61) (Table 2). Proteolytic activity was also exhibited by *P. kudriavzevii* N‐X (mean Hz value of 0.58) and *P. kudriavzevii* 4‐5 (mean Hz value of 0.63) on casein media. No proteolytic activity (Hz value of 1.00) was detected for any of the *K. marxianus* isolates, including isolate N‐9 (Table 2, Figure S2). *Penicillium brevicompactum* was isolated from an artisanal raw ewe's milk cheese in a previous study and a high proteolytic activity of that strain was determined quantitatively (Ozturkoglu Budak, Figge, Houbraken, & de Vries, 2016b). The same primary proteolysis was recently described during ripening of ewe milk Canes trato Pugliese cheese (De Pasquale et al., 2014a,b). Proteolysis during maturation is essential in most cheese varieties, and the catabolism of amino acids obtained by protease hydrolysis leading to the production of a wide array compounds including carboxylic acid, aldehydes, alcohols, thiols and other sulfur compounds, phenols and hydrocarbons (Fox et al., 2017).

Due to substrate specificity, lipolytic enzymes are classified according to the chain length of the hydrolyzed acylglycerols into esterases (EC 3.1.1.1) and lipases (EC 3.1.1.3). Esterases are defined as enzymes that hydrolyze short‐chain acylglycerols (*C* ≤ 10), whereas lipases hydrolyze long‐chain acylglycerols (*C* > 10) (Baur et al., 2015). Tributyrin (C4) is used as a substrate for the majority of screening tests for lipolytic activity.

In this study, two substrates (tributyrin and ROA) were used to screen selected yeast strains for lipolytic enzymes. Almost all of the predominant yeast isolates exhibited extracellular lipolytic activity on tributyrin and ROA media. *Pichia kudriavzevii* N‐X showed the highest extracellular lipolytic activities on both tributyrin (mean Hz value of 0.54) and ROA (mean fluorescent zone (Fz) value of 0.62). *Kluyveromyces marxianus* 5‐7 also had high extracellular lipolytic activity on tributyrin (mean Hz value of 0.60) and ROA media (mean Fz value of 0.70). *K. lactis* and *K. marxianus* isolated from cheese and other dairy foods also displayed lipolytic activities (Binetti et al., 2013). In addition, yeast strains such as *Y. lipolytica* showed high lipase activity and played an important role in the flavor of dairy products (Gkatzionis et al., 2013; Price et al., 2014). Yeast species such as *Candida*,* Kluyveromyces*, and *Pichia* have been reported as good lipase producers isolated from raw milk (Cocolin et al., 2002). Lipases mainly hydrolyze fats and oils to glycerol and fatty acids and have the potential to be used for large‐scale production of commercial cheese (Ozturkoglu‐Budak et al., 2016a). Some short‐chain fatty acids, which have strong and characteristic flavors, are produced through the action of lipases in a process referred to as lipolysis. The fatty acids may be converted to aromatic compounds, especially methyl ketones and lactones, and these methyl ketones can be reduced subsequently to corresponding secondary alcohols (Fox et al., 2017).

### 3.3 Analysis of volatile compounds produced

The aromatic profile from the predominant enzyme producing yeast strains in Hazak cheese‐like medium (HCLM) were determined using HS‐SPME/GC–MS. A number of compounds (33) were detected and included nine alcohols, seven esters, five ketones, six aldehydes, five acids, and four terpenoids (Table 3). Alcohols and esters were the main volatiles identified in the headspace of HCLM.

**Table 3:** Volatile compounds detected in the headspace of CLM medium after exhibiting enzymatic activities of dominant yeast strains growth^a^

No	Volatile components	*Kluyveromyces marxianus*					*Pichia kudriavzevii*			*Kluyveromyces lactis*	
		5‐7	5‐11	N‐9	24‐5	27‐2	4‐5	10‐8	N‐X	NTE	N‐S
Alcohols											
A1	Ethanol	3.115 ± 0.102C	2.889 ± 0.05^C^	1.018 ± 0.027^B^	1.579 ± 0.059^B^	1.436 ± 0.055^C^	0.003 ± 0.001^C^	0.003 ± 0.005^A^	6.519 ± 0.009^A^	6.576 ± 0.032^A^	4.285 ± 0.107^B^
A2	2‐Methylbutanol	0.011 ± 0.003^D^	0.078 ± 0.002^D^	0.290 ± 0.443^D^	0.840 ± 0.062^A^	0.504 ± 0.113^C^	0.263 ± 0.010^D^	1.207 ± 0.030^A^	1.100 ± 0.104^A^	0.573 ± 0.010^B^	0.934 ± 0.066^A^
A3	3‐Methylbutanol	1.207 ± 0.03^A^	0.773 ± 0.045^B^	0.529 ± 0.458^BC^	0.390 ± 0.031^C^	0.083 ± 0.010^D^	nd	0.012 ± 0.003^D^	0.107 ± 0.012^D^	0.124 ± 0.001^D^	0.100 ± 0^D^
A4	2,3‐Butanediol	0.06 ± 0.009^AB^	0.076 ± 0.003^AB^	nd	0.009 ± 0.001^B^	0.000	nd	0.002 ± 0.002^A^	0.000	0.202 ± 0.261^A^	nd
A5	1‐Propanol	0.003 ± 0.005^D^	0.002 ± 0.001^D^	0.835 ± 1.446^CD^	2.583 ± 0.075^A^	2.095 ± 0.030^AB^	1.612 ± 0.065^BC^	1.003 ± 0.135^C^	2.044 ± 0.046^AB^	1.503 ± 0.032^BC^	1.273 ± 0.230^BC^
A6	Phenylethanol	2.27 ± 0.071^AB^	2.518 ± 0.161^A^	1.726 ± 1.331^B^	0.376 ± 0.053^C^	0.177 ± 0.009^C^	0.021 ± 0.002^C^	nd	nd	0.226 ± 0.029^C^	nd
A7	Citronellol	nd	0.22 ± 0.002^A^	0.126 ± 0.109^B^	nd	nd	nd	0.170 ± 0.019^AB^	nd	0.033 ± 0.001^C^	nd
A8	Isopropyl alcohol	nd	0.075 ± 0.004^B^	0.022 ± 0.038^C^	0.006 ± 0.001^C^	nd	nd	0.012 ± 0.004^C^	0.154 ± 0.007^A^	0.074 ± 0.014^B^	0.074 ± 0.015^B^
A9	2‐Methylpropanol	0.099 ± 0.017^E^	0.072 ± 0.009^E^	0.292 ± 0.397^DE^	0.961 ± 0.075^C^	0.533 ± 0.002^D^	0.108 ± 0.033^E^	0.092 ± 0.006^E^	7.034 ± 0.040^A^	1.059 ± 0.012^C^	5.67 ± 0.209^B^
Esters											
E1	Ethyl acetate	0.008 ± 0.001^D^	1.449 ± 0.063^A^	0.51 ± 0.426^B^	0.252 ± 0.067^BC^	0.373 ± 0.295^BC^	0.000	0.020 ± 0.003^D^	0.088 ± 0.003^CD^	0.064 ± 0.010^D^	0.059 ± 0.017^D^
E2	3‐Methylbutyl acetate	0.063 ± 0.012^C^	0.082 ± 0.009^C^	0.437 ± 0.726^BC^	0.089 ± 0.012^C^	0.644 ± 0.016^B^	0.133 ± 0.013^C^	0.148 ± 0.038^C^	0.081 ± 0.009^C^	0.632 ± 0.003^B^	1.762 ± 0.194^A^
E3	Ethyl phenyl acetate	0.129 ± 0.044^BC^	0.531 ± 0.007^AB^	0.86 ± 0.703^A^	0.042 ± 0.003^C^	0.046 ± 0.002^C^	nd	nd	0.133 ± 0.012^BC^	0.212 ± 0.287^BC^	0.133 ± 0.012^BC^
E4	Phenylethyl butyrate	nd	0.068 ± 0.016^A^	0.034 ± 0.022^B^	0.007 ± 0.002^C^	0.006 ± 0.002^C^	0.003 ± 0.001^C^	0.011 ± 0.003^C^	0.006 ± 0.002^C^	0.007 ± 0.001^C^	0.006 ± 0.002^C^
E5	Butyl acetate	0.067 ± 0.008^B^	0.004 ± 0.001^B^	0.031 ± 0.039^B^	0.019 ± 0.001^B^	0.027 ± 0.008^B^	0.023 ± 0.012^B^	0.018 ± 0.003^B^	0.016 ± 0.002^B^	0.284 ± 0.378^A^	0.036 ± 0.004^B^
E6	Ethyl octanoate	0.013 ± 0.002^C^	nd	0.059 ± 0.032^A^	0.046 ± 0.002^AB^	0.045 ± 0.004^AB^	nd	nd	nd	0.034 ± 0.005^B^	nd
E7	Methyl‐2‐methylpropionate	nd	nd	0.015 ± 0.013^B^	nd	nd	nd	nd	0.026 ± 0.001^A^	nd	0.017 ± 0.002^B^
Ketones											
K1	2‐Pentanone	0.185 ± 0.003^A^	nd	nd	nd	nd	nd	nd	0.082 ± 0.010^B^	nd	nd
K2	2‐Heptanone	0.006 ± 0.001^B^	nd	nd	nd	nd	nd	nd	0.026 ± 0.001^A^	nd	0.004 ± 0.004^B^
K3	2‐Nonanone	0.021 ± 0.003^C^	nd	nd	0.163 ± 0.005^A^	nd	nd	0.023 ± 0.011^C^	0.077 ± 0.006^B^	0.082 ± 0.009^B^	nd
K4	Acetoin	0.035 ± 0.004^A^	0.016 ± 0.003^B^	nd	0.004 ± 0.002^D^	nd	nd	0.011 ± 0.003^C^	0.034 ± 0.004^A^	0.009 ± 0.001^C^	nd
K5	4‐Penten‐2‐one	nd	0.016 ± 0.003^C^	0.011 ± 0.020^CD^	nd	nd	0.005 ± 0.002^CD^	nd	0.101 ± 0.002^A^	0.054 ± 0.011^B^	0.017 ± 0.002^C^
Aldehydes											
Q1	Acetaldehyde	0.026 ± 0.006^A^	nd	0.022 ± 0.019^A^	0.005 ± 0.002^BC^	nd	0.007 ± 0.003^BC^	0.004 ± 0.003^BC^	0.017 ± 0.002^AB^	0.003 ± 0.003^C^	0.027 ± 0.010^A^
Q2	3‐Methylbutanal	0.009 ± 0.003^BC^	0.015 ± 0.004^B^	0.006 ± 0.007^CD^	nd	nd	nd	0.015 ± 0.005^B^	0.032 ± 0.004^A^	0.030 ± 0.009^A^	nd
Q3	Benzaldehyde	nd	0.027 ± 0.003^BC^	0.125 ± 0.198^B^	0.026 ± 0.008^BC^	0.019 ± 0.004^BC^	0.040 ± 0.006^BC^	0.078 ± 0.004^BC^	0.086 ± 0.010^BC^	0.238 ± 0.036^A^	0.041 ± 0.009^BC^
Q4	Citral	0.159 ± 0.01^BC^	1.599 ± 0.044^A^	0.239 ± 0.207^B^	nd	nd	0.003 ± 0.001^D^	0.013 ± 0.005^D^	0.166 ± 0.001^BC^	0.111 ± 0.003^CD^	0.01 ± 0.004^D^
Q5	à‐Campholenal	0.147 ± 0.028^A^	0.043 ± 0.012^AB^	0.108 ± 0.187^AB^	0.029 ± 0.002^B^	nd	nd	0.016 ± 0.002^B^	0.014 ± 0.005^B^	0.031 ± 0.006^B^	0.012 ± 0.003^B^
Q6	Hexanal	0.013 ± 0.001^B^	0.019 ± 0.005^B^	0.233 ± 0.202^A^	0.023 ± 0.001^B^	0.033 ± 0.003^B^	0.0123 ± 0.003^B^	0.004 ± 0.005^B^	nd	nd	nd
Q7	2‐Methylpentanal	nd	nd	0.1283 ± 0.222^DE^	0.415 ± 0.008^C^	0.084 ± 0.007^DE^	0.181 ± 0.054^D^	0.157 ± 0.068^D^	2.801 ± 0.003^A^	0.930 ± 0.035^B^	0.211 ± 0.074^D^
Acids											
S1	Acetic acid	1.004 ± 0.067^A^	0.407 ± 0.020^B^	0.383 ± 0.035^B^	nd	0.002 ± 0.003^C^	0.016 ± 0.002^C^	0.032 ± 0.007^C^	0.029 ± 0.009^C^	0.028 ± 0.009^C^	nd
S2	3‐Methylbutanoic acid	nd	0.002 ± 0.003^B^	0.235 ± 0.198^A^	0.084 ± 0.010^B^	0.003 ± 0.005^B^	0.001 ± 0.002^B^	nd	0.007 ± 0.002^B^	0.006 ± 0.005^B^	0.001 ± 0.001^B^
S3	Octanoic acid	0.008 ± 0.002^A^	0.002 ± 0.002^AB^	0.042 ± 0.062^A^	0.024 ± 0.001^AB^	0.003 ± 0.006^AB^	0.020 ± 0.003^AB^	0.017 ± 0.002^AB^	0.037 ± 0.016^AB^	0.039 ± 0.007^AB^	0.027 ± 0.003^AB^
S4	Propanoic acid	0.045 ± 0.011^B^	0.016 ± 0.002^B^	0.103 ± 0.087^A^	0.011 ± 0.004^B^	0.010 ± 0.001^B^	0.035 ± 0.003^B^	nd	0.014 ± 0.005^B^	0.015 ± 0.003^B^	0.010 ± 0.004^B^
Terpenoids											
T1	(E,Z)‐à‐Farnesene	0.005 ± 0.001^BC^	0.011 ± 0.004^BC^	0.005 ± 0.005^BC^	nd	nd	nd	nd	0.047 ± 0.062^B^	0.046 ± 0.002^B^	0.002 ± 0.004^C^
T2	Limonene	0.016 ± 0.002^A^	nd	nd	0.016 ± 0.015^A^	nd	nd	0.015 ± 0.004^A^	0.02 ± 0.035^A^	0.013 ± 0.001^A^	nd
T3	1,4‐Pentadiene	nd	0.009 ± 0.009^AB^	0.003 ± 0.006^BC^	0.002 ± 0.003^BC^	nd	nd	0.002 ± 0.003^C^	0.011 ± 0.003^C^	nd	0.011 ± 0.003^A^
T4	7‐methyl‐1‐Octene	0.026 ± 0.008A	0.001 ± 0.002^B^	nd	nd	nd	nd	nd	nd	nd	nd

nd, Not detected.

Values in the same row bearing different letters differ significantly (*p* < .05).

a Data are means of three replicates. Values expressed as mean relative concentration (compound area/internal standard area × 0.0555mg/ml) ±standard deviation of the main volatile compounds.

Among alcohols, the most abundant volatiles were ethyl alcohol, followed by phenyl ethanol and 3‐methylbutanol. And 2, 3‐butanediol, 2‐methylpropanol, isopropyl alcohol, citronellol were also found from some selected strains (Table 3). The formation of alcohols that encountered in cheese involve in methyl ketone and aldehydes reduction, lactose metabolism, amino acid metabolism as well as degradation of linoleic and linolenic acids (Spinnler & Molimard, 1996).

With regard to esters, ethyl acetate, 2‐phenethyl acetate and 3‐methylbutyl acetate were extensively produced from most *P. kudriavzevii*,* K. lactis*, and *K. marxianus* strains (Table 3). Esters are common cheese volatiles from esterification reactions between short‐ to medium‐chain fatty acids and primary and secondary alcohols (Delgado, González‐Crespo, Cava, & Ramírez, 2011). Most esters encountered in cheese are described as having sweet, fruity, and floral notes. Especially ethyl acetate could contribute to the aroma of cheese by minimizing the sharpness and bitterness derived from carboxylic acids (Curioni & Bosset, 2002).

Acetic acid was the most abundant acid detected, while propanoic acid, 3‐methylbutanoic acid, and octanoic acid were also produced by some strains. During the ripening of cheeses, carboxylic acids can be originated from three main biochemical pathways: (1) lipolysis (hydrolysis of triglycerides into free fatty acids, such as butanoic, pentanoic, hexanoic, heptanoic, octanoic, nonanoic, decanoic and undecanoic acids); (2) proteolysis (breakage of caseins into peptides and amino acids, such as 2‐methylpropanoic and 3‐methylbutanoic acids); and (3) lactate metabolism (acetic and propanoic acids) (Delgado et al., 2011). Acetic acid contributes to the typical flavor of most types of cheese, giving rise to vinegar‐like, peppery, fruity, and floral odors. However, although aldehydes and terpenoids were not major components, they may also play an important role in the flavor in these products (Table 3).

**Figure 1:**
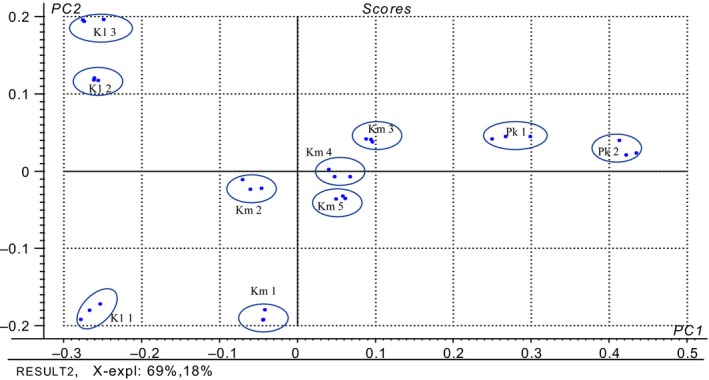
Loadings of the principal components (PC 1‐PC 2) of the analyzed parameters (volatile compounds) of liquid mimicking cheese medium after growth of different yeast strains: *Kluyveromyces marxianus* (5‐7, 5‐11, N‐9, 24‐5, and 27‐2), *Kluyveromyces lactis* (N‐E, N‐S), *Pichia kudriavzevii* (4‐5, 10‐8 and N‐X). All analyses were analyzed in duplicate

**Figure 2:**
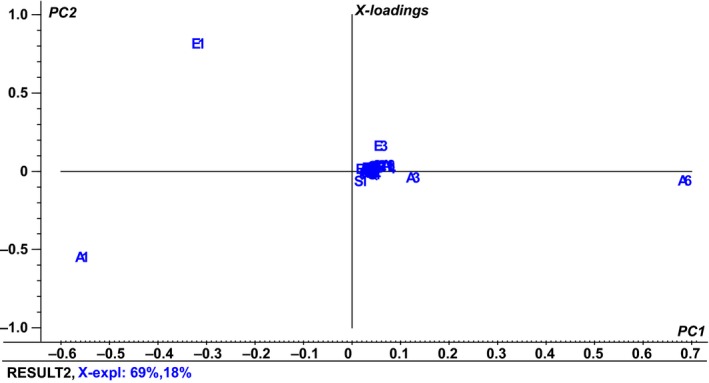
Score plot of PC 2 versus PC 1 for the principal components of volatile compounds (Alcohols A 1‐A 9; Esters E 1‐E 7; Ketones K 1‐K 5; Aldehydes Q 1‐Q 7; Acids S 1‐S 5; Terpenoids T1–T4 in table 3) of different yeast strains: *K. marxianus* (5‐7, 5‐11, N‐9, 24‐5, and 27‐2), *K. lactis* (N‐E, N‐S), *P. kudriavzevii* (4‐5, 10‐8, and N‐X). Principal Component Analysis was performed using Unscramble software (V.9.7, CAMO ASA, and Oslo, Norway)

Principal component analysis was carried out in order to interpret the relationship between composition of the volatile compounds and the different yeast strains. Results from the first two PCA could explain 69% and 18% variances, respectively (Figure 1). The difference between selected yeasts and volatile compounds produced could be easily distinguished by the concentration of ethanol (A1), ethyl acetate (E1), phenyl ethanol (A6), and 2‐phenethyl acetate (E3), 3‐methylbutanol (A3), and acetic acid (S1) (Figure 2).

**Figure 3:**
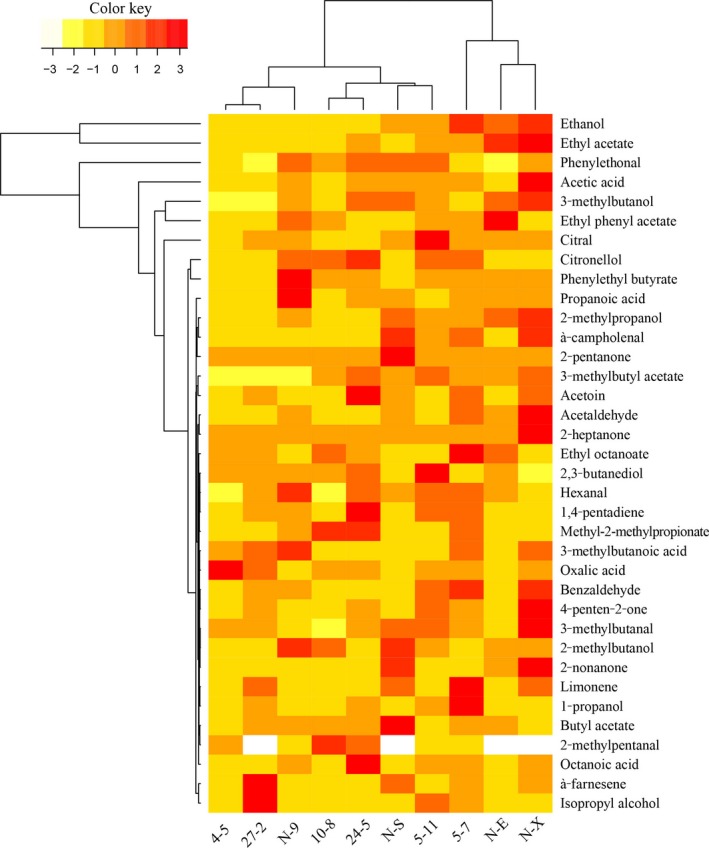
Comparison between concentrations of volatile compounds (Alcohols A1–A9; Esters E1–E7; Ketones K1–K5; Aldehydes Q1–Q7; Acids S1–S5; Terpenoids T1–T4 in Table 3) and enzyme‐producing of selected yeast strains: *K. marxianus* (5‐7, 5‐11, N‐9, 24‐5, and 27‐2), *K. lactis* (N‐E, N‐S), *P. kudriavzevii* (4‐5, 10‐8, and N‐X) in Cheese Like Medium. Permutation analysis of volatile components were used for clustering by R Project (R version 3.3.2). The colors correspond to normalized mean data levels from low (white) to high (yellow)

The heat map was used to visualize details of 36 flavor compounds produced (Figure 3). Most of *P. kudriavzevii*,* K. marxianus*, and *K. lactis* strains were good producers of flavor compounds. Interestingly, most of the key compounds were associated with the predominant proteolytic and lipolytic yeasts, such as *P. kudriavzevii* N‐X, *K. lactis* N‐S, and *K. marxianus* 5‐7 (Table 2). *P. kudriavzevii* N‐X showed the greatest production of the main volatile compounds such as ethyl acetate, 3‐methylbutanol and acetic acid.

Moreover, *K. marxianus* strains were associated with the production of ethanol, phenyl ethanol and 3‐methylbutanol. Phenyl ethanol is among the most cheese with pleasant rose flower notes. Strecker degradation from phenylalanine is also responsible for 2‐phenylethanol production and this reaction seems to be essentially metabolized by yeasts (Nogueira, Lubachevsky, & Rankin, 2005). The presence of branched‐chain primary alcohols, such as 3‐methylbutanol, indicates the reduction in 3‐methylbutanal produced from leucine. It was identified in bovine Mozzarella and other goat cheese (Castillo, Calvo, Alonso, Juárez, & Fontecha, 2007; Curioni & Bosset, 2002).

The present research demonstrates that *K. marxianus* 24‐5 had the ability to decompose lactose and produce aroma compounds such as phenyl ethanol and 3‐methylbutanol, and may have the potential to be used in the industrial manufacture of cheese and other low‐lactose dairy products. However, *P. kudriavzevii* 4‐5 and *P. kudriavzevii* 10‐8 were also related to the presence of volatile compounds, such as 2‐pentanone and 2‐heptanone, and these aroma compounds have been shown to contribute to cheesy flavor in blue cheese (Martin, Berger, & Spinnler, 2002).

However, the contribution of yeasts to flavor during cheese ripening is generally underestimated (Fox et al., 2017). Metabolic pathways involving the synthesis of aroma and flavor molecules are linked to the action of modifying enzymes in the cell. This study offers an opportunity to exploit *K. marxianus* as a new organism for the production of flavor compounds in cheese (Carlquist et al., 2015; Morrissey et al., 2015; Pires, Teixeira, Branyik, & Vicente, 2014).

The acetate esters (ethyl acetate, 2‐phenethyl acetate, and 3‐methylbutyl acetate) are derived from alcohol groups (ethanol, phenyl ethanol, and 3‐methylbutanol), acetate (in the form of acetyl‐CoA), medium chain fatty acids (MCFA) and ethyl esters (ethyl hexanoate and ethyl octanoate). The formation of these esters is dependent on the concentration of the substrates acyl‐CoA and alcohol, and the activity of the enzymes involved in the pathways (Cordente, Curtin, Varela, & Pretorius, 2012).

In this study, the relative abundance of acetic acid esters (ethyl acetate, 2‐phenethyl acetate, 3‐methylbutyl acetate) were significantly higher in *P. kudriavzevii* N‐X than in other species, and the mean relative concentration of ethyl acetate detected in *P. kudriavzevii* N‐X was approximately 7.016 g·L^−1^. The high proteolytic and lipolytic activities of *P. kudriavzevii* N‐X play a key role in the formation of esters (Table 2, Figure 3). Other researchers have reported that ethyl acetate and 3‐methylbutyl acetate were the main esters produced by *Kluyveromyces* yeasts (Padilla et al., 2014; Price et al., 2014). 2‐phenethyl acetate is one of the most important aromatic esters providing a floral odor to Camembert cheese. The branched esters 3‐methylbutyl acetate also plays an important role in the aroma of Emmental cheese (Curioni & Bosset, 2002), and the sensory characteristics of bovine Mozzarella cheese seem to depend primarily on 3‐methylbutyl acetate and ethyl isobutanoate (Kubícková & Grosch, 1998).

Another important characteristic flavor in Kazak cheese, was due to 3‐methylbutanol which was highly prevalent in the HCLM of predominant yeasts. One amino acid obtained by protease hydrolysis can be converted to a range of volatile compounds. Leucine can be converted to 2‐keto‐4‐methylpentanoic acid through aminotransferase, and formed 3‐methylbutanal after decarboxylation, and 3‐methylbutanol through reduction (Fox et al., 2017).

## 4 CONCLUSIONS

Cheese ripening involves a series of complex biochemical reactions catalyzed by enzymes produced by microorganisms. This study examined the correlation between the enzymatic abilities of secreted by selected yeasts of Kazak artisanal cheeses and the production of flavor compounds. *Pichia kudriavzevii* was the most predominant yeast species isolated from Kazak artisanal cheeses, followed by *K. marxianus* and *K. lactis*. The generation of aroma compounds (ethanol, ethyl acetate, 3‐methylbutanol, and acetic acid) by *P. kudriavzevii* N‐X, could lead to the formation of characteristic flavors in traditional cheese. The native strains studied in this research could be selected as potential starter or adjunct cultures for the production of industrial cheeses to improve the sensory properties while protecting the traditional character of Kazak artisanal cheeses.
