# Supplementary material for: Potential characterization of yeasts isolated from Kazak artisanal cheese to produce flavoring compounds
Source: Microbiologyopen. 2017 Dec 26;7(1):e00533. doi: 10.1002/mbo3.533 (PMC5822340; doi:10.1002/mbo3.533)
Supplement: Supplementary file 1 [file MBO3-7-na-s001.docx]

**Supplemental Information for**

Potential characterization of yeasts isolated from Kazak artisanal cheese to produce flavoring compounds

**This file includes:**

Supplementary figures (Figure S1、S2)

**Supplementary figure legends**

**Fig. S1. Cheese samples from Ili Kazak Autonomous Prefecture, Altay area, Mulei Kazakh Autonomous County and Barkol Kazak Autonomous County of Uighur Autonomy Region in China**

**
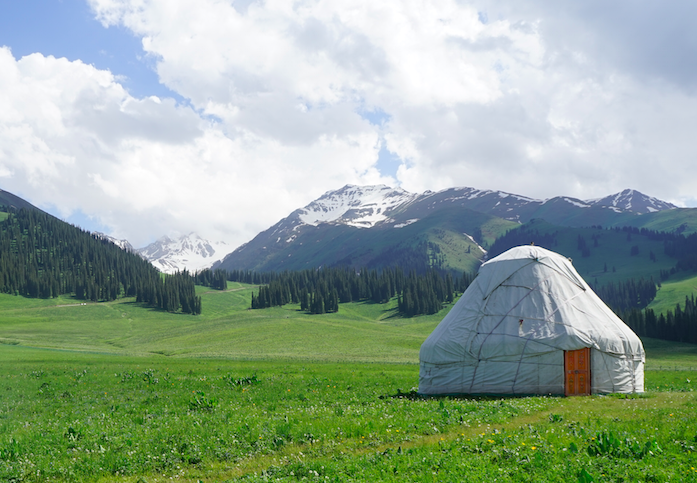

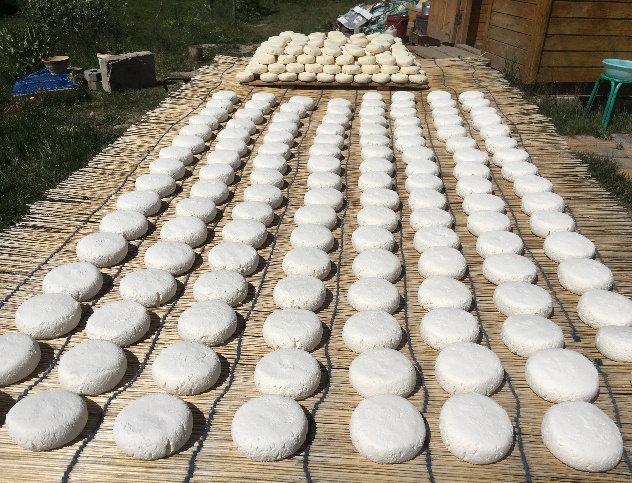
**

**A B**

**
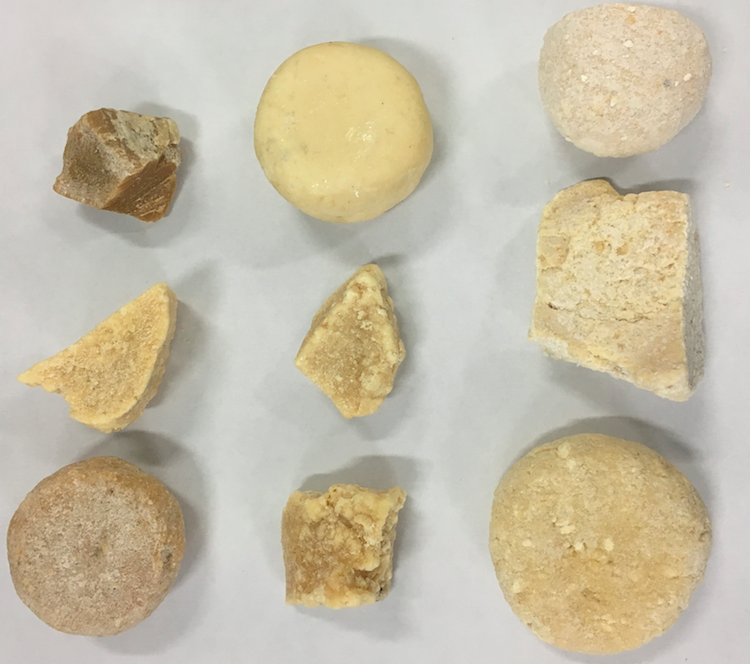

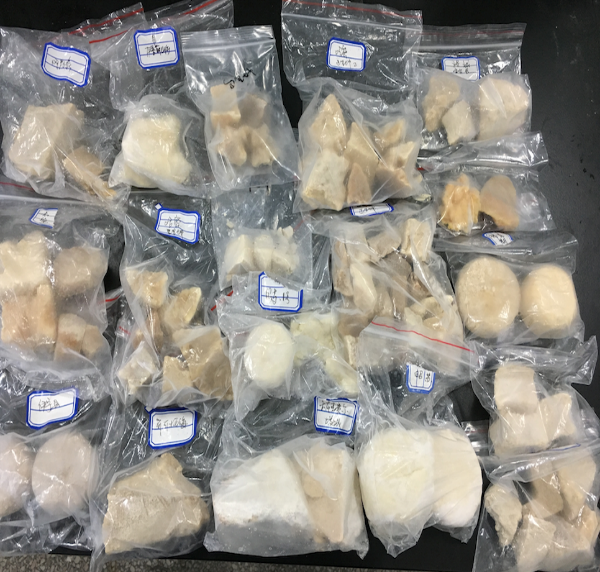
**

C D

**Fig. S1.** Place of Kazak nomads reside in Nalat grasslands of Ili area (A), Cheese in the ripening from Zhaosu country of Ili Kazak Autonomous Prefecture in Xinjiang province, China. **(B)**, Varieties of Kazak cheese from different regions **(C),** and different shapes of cheese samples **(D)**.

**Fig. S2. Preliminary screening for the ability to produce enzymes (protease, lipase and β-galactosidase) by selected yeast strains**

**
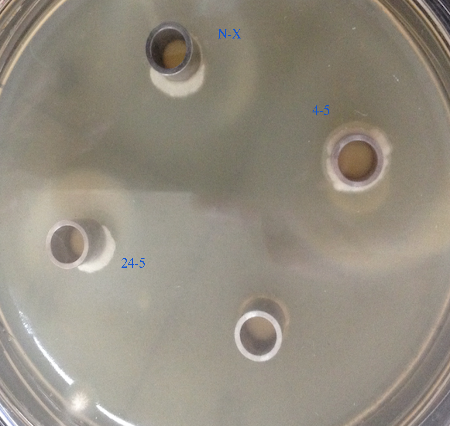

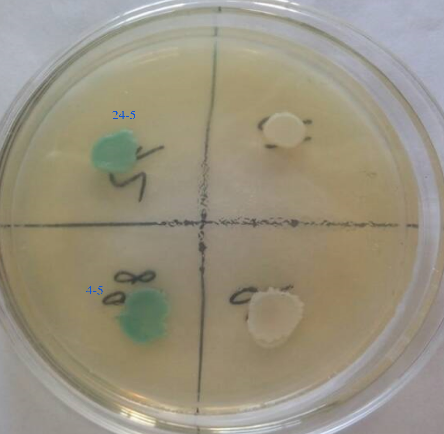
**

**(A) (B)**

**
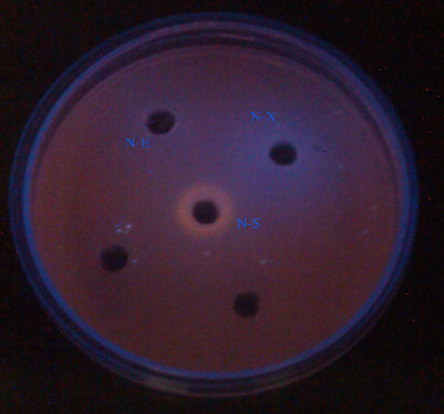

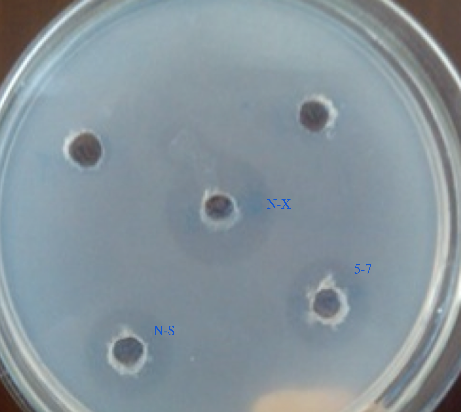
**

**(C) (D)**

Fig. S2. The ability to degrade protein was assessed on YPD agar containing 20 gL-1 of casein solution(A); To screen β-galactosidase activity of YPD plates supplemented with 0.1 mg/ml X-gal, the positive colonies turned blue (B). Screening for the extracelullar lipolytic activities screened on Rodamin B medium (C) and 1% (w/v) tributyrin agar (D). In Fig.S2, N-X: *P. kudriavzevii* N-X; 24-5: *K. marxianus* 24-5; N-S: *K. Lactis* N-S; 4-5: *P. kudriavzevii* 4-5; N-E: *K. Lactis* N-E; 5-7: *K. marxianus* 5-7.
